# Dental Student Perceptions of Distance Education over Time: A Mixed-Methods Study

**DOI:** 10.3390/dj11100233

**Published:** 2023-10-02

**Authors:** Susanna Y. Yeh, Nithya Puttige Ramesh, Kristie Kaczmarek-Stewart, Chiho Ahn, Alice Z. Li, Hiroe Ohyama

**Affiliations:** 1 General Practice Residency in Dentistry, Massachusetts General Hospital, Boston, MA 02114, USA; susanna_yeh@hsdm.harvard.edu; 2 Advanced Graduate Education Program in Dental Public Health, Department of Oral Health Policy and Epidemiology, Harvard School of Dental Medicine, Boston, MA 02115, USA; nramesh@hsdm.harvard.edu; 3 Advanced Graduate Program in Endodontics, University of Pennsylvania School of Dental Medicine, Philadelphia, PA 19104, USA; ksk@upenn.edu; 4 Department of General Dentistry, Section of Predoctoral Periodontology, Boston University Henry M. Goldman School of Dental Medicine, Boston, MA 02118, USA; chahn28@bu.edu; 5 Advanced Graduate Program in Endodontics, Department of Restorative Dentistry and Biomaterials Sciences, Harvard School of Dental Medicine, Boston, MA 02115, USA; alice_li@hsdm.harvard.edu; 6 Department of Restorative Dentistry and Biomaterials Sciences, Harvard School of Dental Medicine, Boston, MA 02115, USA

**Keywords:** dental education, distance education, COVID-19, dental students, a mixed-method, hybrid learning, survey

## Abstract

Objectives: Amid the COVID-19 pandemic, the transition to distance learning raised pertinent questions regarding advantages and challenges compared to traditional in-person learning. This study aimed to investigate dental students’ perceptions of distance learning throughout the pandemic, examining its impact on their education. Methods: This study employed a convergent mixed-methods design. Three online surveys were conducted in 2020, 2021, and 2022 to collect quantitative data. Additionally, qualitative semi-structured interviews were carried out in 2022. Interviews were recorded and transcribed; then, thematic analysis was performed following an inductive approach. Results: As perceived by the participants, distance learning entails advantages and challenges. Initially, they felt uncertainty and negativity about the new environment with distance learning. However, their perceptions shifted positively as they adapted, even after returning to hybrid and in-person modules. Furthermore, most participants felt that distance learning is better suited for didactic content. It was suggested that didactic courses contain approximately 25–50% of online methods. Conclusions: Distance learning has provided valuable opportunities to reinforce curricula and improve learning efficacy during the pandemic. Our findings suggest that a hybrid learning model that combines traditional and distance modules appears to be an effective approach for future dental education.

## 1. Introduction

Distance learning is the educational process in which physically separated students and instructors engage in the exchange of knowledge and information using digital technologies [1]. In recent years, technological advancements have made distance learning more reliable and achievable, leading to its widespread use as an educational model [2,3]. With the necessitated transition to online learning environments during the COVID-19 pandemic, many students and faculty have adapted to the new educational reality [4]. In response, most dental schools in the USA transitioned their didactic curriculum exclusively to online formats during the early stage of the pandemic in 2020. These online classes were often held via group meeting platforms such as Zoom (Zoom Video Communications Inc., San Jose, CA, USA), allowing students to interact through a microphone, chat box, polls, or annotations on a shared screen. As the pandemic evolved, so did the learning environment; when schools were allowed to return to in-person learning, many adopted a hybrid model, where some lectures, discussions, or group activities were held online, and others were in-person. 

The transition to distance learning has posed various questions concerning its benefits and challenges in comparison to traditional in-person learning. In previous studies, students have noted that social, environmental, and technological factors impacted their educational experience in both positive and negative ways [5,6,7,8,9]. While some studies have indicated adverse effects of distance learning on academic performance [10,11,12], others have demonstrated that it may have maintained the quality of student learning [13,14,15,16]. Interestingly, distance learning may be more likely to impact the academic performance of lower-grade college students (i.e., first- and second-year students) in contrast to third- and fourth-year students, as well as postgraduate students [17]. Dental students’ satisfaction with distance learning has been diverse [18,19]. Several adverse effects of distance learning have been reported, including reduced engagement, learning [20,21], and student-instructor interactions [22]. On the other hand, some studies have highlighted students’ positive attitudes towards distance learning during the pandemic [23,24]. Zheng et al. reported that students had a favorable attitude toward online learning and wanted to continue with some online instruction in their future curriculum [15].

A few studies investigated dental students’ perceptions of distance learning and followed them over time, starting from the early phase of the pandemic evolution. The sudden transition to distance learning initially raised concerns, but as students and faculty gained more experience with this format, the implementation of distance learning underwent changes [25]. By studying students’ perceptions of distance learning throughout the COVID-19 pandemic, we can better understand the ways that students adapt to changes in learning environments. This can help identify effective approaches that will transform the post-pandemic and future teaching and learning environments into more optimal educational experiences. 

The purpose of our study was to investigate dental students’ perceptions of distance learning throughout their experience in the COVID-19 pandemic’s evolution. This study may illuminate ways distance learning can be optimally integrated into dental curriculums and enhance teaching and learning post-pandemic and beyond, as our experience during the pandemic may continue to influence the educational approach to dentistry in subsequent years. 

## 2. Materials and Methods

### 2.1. Research Design

This study was conducted at the Harvard School of Dental Medicine (HSDM) in Boston, MA, USA, and was determined exempted by the Harvard Faculty of Medicine, Institutional Review Board (IRB #20-2134). An explanatory sequential design mixed-method study was adopted [26], consisting of three surveys conducted over the course of two years, along with a series of qualitative semi-structured interviews. Qualitative interviews were conducted to gather in-depth responses from diverse participants to further explore key results gathered from the surveys.

### 2.2. Distance Education Modules

At HSDM, prior to the COVID-19 outbreak, all predoctoral students attended didactic sessions in person. Most classes were not recorded and were taught in a large-group setting, with the integration of small-group discussions. In response to COVID-19, all students switched to complete distance learning in March 2020. During the 2020–2021 academic year, students in years 3 and 4 returned to resume clinical activities in a limited capacity while continuing all didactic courses remotely. During the 2021–2022 academic year, all students transitioned to a hybrid format for didactic courses. As the University’s responses to COVID-19 evolved, many didactic courses used both in-person and distance learning, with some sessions being held completely remotely and others being held completely in-person, to adhere to social distancing requirements. The primary digital platform used was Zoom v 5.12 (Zoom Video Communications Inc. San Jose, CA, USA). Most remote didactic sessions were held in real-time, while lecturers were also encouraged to be recorded or pre-recorded.

### 2.3. Data Collection 

#### 2.3.1. Surveys 

Three anonymous surveys were conducted between May 2020 and May 2022. Each survey’s questions were constructed with statistical consultation. The surveys consisted of both close-ended and open-ended questions in English, using a Likert scale ranging from 1 to 5. The first survey in May 2020 was administered to the Class of 2022 (n = 39) through a Zoom poll (Zoom Video Communications Inc. San Jose, CA, USA) at an optional feedback session. This initial survey focused on evaluating students’ experiences and perceptions during their first didactic course conducted through Zoom. It assessed various aspects, such as burnout, engagement, and impacts on retention of materials. Additionally, students were asked about their preference for lecture formats. Sample questions include the following: (i) Distance, compared to in-person, learning has affected my level of burnout in the following way; (ii) Distance, compared to in-person, learning has affected my retention of class material in the following way; (iii) Distance, compared to in-person, learning has affected my intellectual engagement in class in the following way. 

To improve the sample size and gain deeper insights into students’ experiences with distance education, we extended the invitation for the second and third surveys to more classes that had participated in distance learning. The second survey was conducted through Qualtrics (Qualtrics LLC, Provo, UT, USA) from March to June 2021, during which students from the Classes of 2022, 2023, and 2024 were invited to participate by an informational email. The final survey, disseminated through email and conducted via Qualtrics in May 2022, was open to students from the Classes of 2022, 2023, 2024, and 2025. To facilitate meaningful comparisons, the questions across all three surveys were kept nearly identical. For the second and third surveys, we incorporated some questions related to future dental curricula to gather students’ suggestions. Examples of those additional questions include the following: (i) How effective do you believe distance learning would be in the post-pandemic era? (ii) Which participation method would be more effective in distance learning? (iii) What percentages of didactic curriculum should be taught via distance learning platforms in the post-pandemic era? To improve the response rates, three reminder emails for the second and third surveys were sent every two weeks. Only the completed survey results were included in the data analysis.

#### 2.3.2. Semi-Structured Interviews

The qualitative interview series was conducted in February and June 2022. A semi-structured interview guide was developed from the information gathered in the quantitative surveys and collaboratively reviewed and refined by the research team. Classes of 2022, 2023, 2024, and 2025 were invited to participate through email. Interested students were asked to complete a demographic survey, which collected information on the students’ current year in dental school, age, gender, and race/ethnicity. This information was de-identified, and the demographic information was utilized to select a diverse and representative group of participants. Interview questions were open-ended to allow participants to freely express their thoughts and ideas. Examples of questions asked include the following: (i) What are some features of online or in-person learning you found most useful? (ii) Given the courses you have taken so far, are there any you feel are well-suited for online learning? (iii) Have your study habits changed as a result of virtual learning? These were followed up with probing questions to obtain detailed information. All interviews were conducted one-on-one via Zoom v 5.12. At the beginning of each interview, informed consent was obtained. Interviews averaged 30–45 min each and were audio-recorded with the participant’s consent. To maintain anonymity, participants were instructed to assign their Zoom name to their Subject ID and disable their camera. Data collection was stopped when thematic saturation was achieved with 11 interviews, of which there were no dropouts [27,28]. To incentivize participation, survey and interview participants were given a USD 5 and USD 30 electronic gift card, respectively.

### 2.4. Data Analysis

The results of the surveys were descriptively analyzed using Qualtrics Stats iQ™. For the qualitative data, Rev (Rev Transcription Services, San Francisco, CA, USA) was used to create transcripts of the interviews. Thematic analysis was performed using a mixed deductive (directed content analysis) and an inductive (grounded theory) approach to analyze data [29]. One author (N.P.R.) applied pre-determined codes based on the survey data and the interview guide with additional inductive codes generated from the data through an iterative consensus-building approach after coding the first three transcripts [30]. These were reviewed by three other authors (S.Y., K.K., and H.O.) and discussed to achieve consensus. Coding changes were tracked, and inductive thematic saturation was assessed by noting when no additional codes were added [28]. After the completion of coding of all the transcripts, the relationship between the extracted codes was determined, and subthemes and overarching themes were assigned. We drew on the perspectives of four authors (N.P.R., S.Y., K.K., and H.O.) to synthesize overarching themes and to facilitate a shared understanding of the data. In case of disagreements among analysis team members over the coding, a discussion was conducted until a consensus was achieved. The Standards for Qualitative Research Reporting guidelines [31] were used as a guide to improve our methodological quality and reporting standards. Mixed methods results were synthesized using data triangulation methods described by [32]. We used methodological triangulation by comparing results across our quantitative and qualitative findings. Investigator triangulation was achieved by having at least two of the same researchers in both data collection and analyses, and other research team members were also involved in the review and discussion of findings across our methods.

## 3. Results

### 3.1. Quantitative Data

The three survey response rates were 100.0% (n = 39/39), 24.3% (n = 25/103), and 21.2% (n = 29/137), respectively, from first to last. Table 1 presents the participant’s overall characteristics and response rate of each survey.

#### 3.1.1. Students’ Preferences for Distance Education Modules

**Figure 1:**
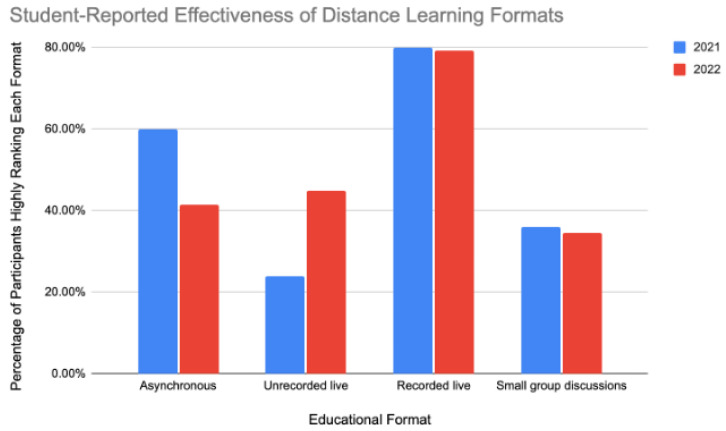
Student-reported effectiveness of distance learning format. Bar chart representing two surveys conducted in 2021 (n = 25) and 2022 (n = 29) where students ranked four formats of distance learning. The *x*-axis represents the class formats, and the *y*-axis represents the percentage of students who gave a high ranking (1st or 2nd) to each format.

Effectiveness of class formats: In the 2021 and 2022 surveys, participants were asked to rank 4 distance learning formats in order of preference: (1) recorded live lectures, (2) non-recorded live lectures, (3) asynchronous lectures, and (4) small group discussions, The respondents rated the class formats from 1st (most effective) to 4th (least effective). Rankings of “1st” or “2nd” were recorded as “highly ranked,” while rankings of “3rd”, or “4th” were “lowly ranked”. Recorded live lectures were consistently the most preferred format in both surveys (80.00% in 2021 and 79.31% in 2022). However, there was also a shift in preferences between 2021 and 2022. Non-recorded live lectures gained popularity, while asynchronous lectures became less preferred (Figure 1).

#### 3.1.2. Students’ Experiences with Distance Learning

**Figure 2:**
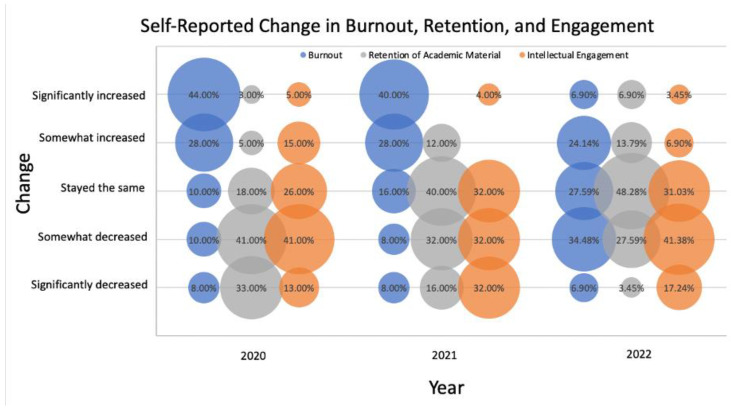
Student-Reported change in burnout, retention, and engagement. Bubble plots for the responses to three questions that assess participants’ self-reported changes in burnout, retention of academic material, and intellectual engagement compared to pre-pandemic in-person learning. Bubble size is the percentage of participants who selected each answer choice. 2020 (n = 39), 2021 (n = 25), and 2022 (n = 29).

Burnout (Emotional exhaustion): In the 2020, 2021, and 2022 surveys, students were asked to compare their experience with distance learning to pre-pandemic in-person learning. Figure 2 shows the impact of distance learning on students’ levels of burnout. During the early pandemic (2020 and 2021), most participants (71.79% and 68.00%, respectively) reported increased burnout compared to their pre-pandemic learning experiences. However, the levels of burnout showed a decline when the students were surveyed again in 2022. In 2022, only 31.03% of participants felt their burnout had increased, while 41.38% of participants felt their burnout had decreased. 

Retention of Academic Material: In the early pandemic year of 2020, most participants (74.00%) reported a decrease in retention of class material compared to their pre-pandemic in-person learning experiences. Over time, the percentage of students reporting a decrease in retention steadily declined to 48.00% in 2021, with 16.00% reporting a significant decrease, and in 2022 further decreased to 31.03%, with only 3.00% reporting a significant decrease. Moreover, the percentage of students who reported no change in retention of class material increased from 18.00% in 2020 to 48.00% in 2022 (Figure 2). 

Intellectual Engagement: Most participants reported that their intellectual engagement with academic material either significantly or somewhat decreased compared to their pre-pandemic learning experiences (54.00% in 2020, 64.00% in 2021, and 58.62% in 2022). This trend remained consistent throughout the three time periods (Figure 2).

#### 3.1.3. Students’ Suggestions 

**Figure 3:**
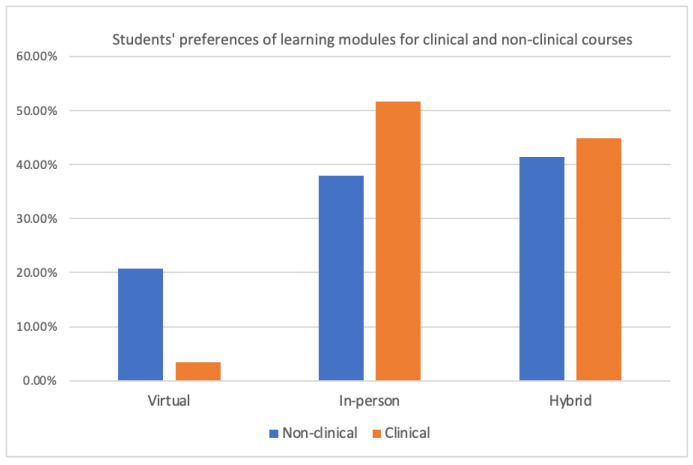
Students’ preferences of learning modules for clinical and non-clinical courses. Bar chart representing a survey result conducted in 2022 (n = 29) where students were asked about their learning module preferences for clinical and non-clinical courses. The *x*-axis represents the learning modules, and the *y*-axis represents the percentage of students’ preference for each module.

Distance Learning Post-Pandemic and Beyond: In the 2021 and 2022 surveys, participants were asked about their opinions of distance learning during the pandemic. Most participants (75.00% in 2021 and 65.52% in 2022) believed that distance learning would be effective in the post-pandemic era. When students were asked about their learning module preferences for clinical and non-clinical courses in 2022, it was revealed that more students favored hybrid models for both types of courses, with 41.38% favoring non-clinical and 44.83% for clinical courses. Interestingly, most students (51.72%) still showed a preference for an in-person learning model specifically for clinical courses, while 37.93% favored the same for non-clinical courses, as illustrated in Figure 3.

**Figure 4:**
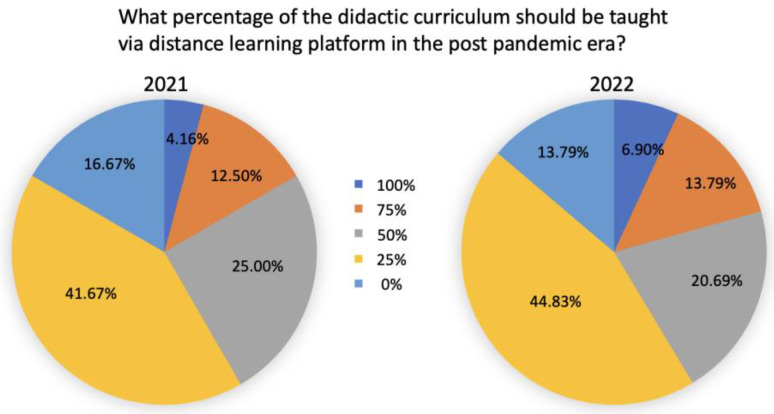
Optimal percentage of distanced didactic teaching in the post-pandemic era. Two pie charts representing participants’ opinions on the percentage of didactic curriculum that should be taught via distance learning formats in the post-pandemic era. 2021 (n = 25) and 2022 (n = 29).

Participants were also asked what percentage of didactic curriculum should be taught via distance learning platforms in the post-pandemic era. The most preferred option was 25% of the didactic curriculum, with 41.67% of participants in 2021 and 44.83% in 2022 indicating this preference. The second preference was 50%. More than half of students (66.67% in 2021 and 65.52% in 2022) preferred to have between 25% to 50% of the didactic curriculum taught via distance learning modules (Figure 4).

**Figure 5:**
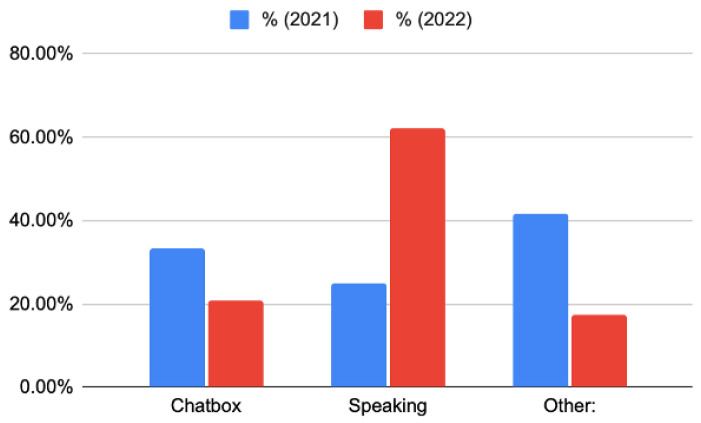
Students’ preferences on participation methods during the distance learning sessions. Bar chart representing two surveys conducted in 2021 (n = 25) and 2022 (n = 29) where students were asked about their preferences on participation methods during distance didactic lectures. The *x*-axis represents the participation methods, and the *y*-axis represents the percentage of students’ preference for each method.

Additionally, participants were asked to indicate the most effective participation method during remote didactic lectures. Commonly used methods include speaking through the microphone and utilizing a chat box, while alternative methods may include annotating on a shared screen and posting on a discussion forum. In 2021, the responses were diverse, with 33.33% of respondents selecting the chat box, 25.00% opting for speaking, and 41.67% indicating “other” as the most effective method. Participants elaborated on “other” to include methods such as writing on an interactive whiteboard, participating in quizzes, and balancing both written chats and speaking. There was a noticeable shift in 2022, as two-thirds of the participants (62.07%) believed speaking was the most effective participation method. (Figure 5).

Which participation method would be more effective in distance learning?

### 3.2. Qualitative Data and Themes 

The interviews were used as a tool to obtain more in-depth responses regarding some of the findings from the surveys. Table 1 provides an overview of the academic years of the interview participants. The collected data were carefully coded and grouped into three overarching themes. They were used to explore the students’ preferred modalities, their inputs on factors impacting their learning experiences, and the barriers and facilitators for distance learning. The detailed descriptions of the themes are presented as follows.

**Theme 1.**  *The type of content influenced the student’s preferred modality.*

Our survey results showed that students had positive perceptions towards both in-person as well as online lectures. Similarly, many interview respondents reported liking remote lectures, while some respondents preferred in-person lectures. A recurrent theme in all interviews was that the subject of the class heavily influenced their choice of in-person versus distance learning. Students preferred distance formats for didactic lectures on non-clinical subjects but in-person if there was a clinical component to the material. Participants agreed that a mix of both modalities based on the content would be the most favorable option.

*“I feel like visual learning is the key element. So, pathology, you have a slide. And so, with our screen, it just works well. It’s not going to change that much. Visual learning, it’s online, I like it. It’s not a bother. But then, when it comes to the clinical aspect of it, there’s just so many reasons I prefer in-person. For ortho and prosth, I need to visualize things. I like to be able to interact with the professor for clinical courses, and it’s just more organic and easier.”*

Some students reported that their learning styles were not impacted, as the style of examination and grading systems remained the same. Like our survey results, others noted that attending classes online and having access to recorded lectures made it more convenient and effective to revisit course content and take comprehensive notes.

*“when I would go to class, I thought I was learning, but just hearing something wasn’t enough. So, when class finished and I wanted to study, review, do homework, I wouldn’t go back to a lecture again. But now I have the [recorded] lecture, the actual words that they said, the examples that I can go back on. So, I think I listen to lectures more carefully now, even if it’s the first time through. I guess I’m more attentive because I can pause, process, and then restart or play again.”*

**Theme 2.**  *Appropriate use of technology and teaching tools helped facilitate learning in any modality.*

Participants highlighted several ways in which technology and teaching tools can be utilized to improve their learning experience in both remote and in-person modalities. During remote lectures, participants found several enhancements that positively impacted their learning, such as breakout rooms, polling, and annotating features. On the other hand, in-person lectures were perceived as more engaging when instructors effectively utilized the physical space and interacted with the students. Some participants reported further that using computers and note-taking devices during in-person classes had accustomed them to using technology for educational purposes, thus aiding their learning process in both formats.

*“I definitely think in-person has more engagement in the audience just because you can get a little bit more feel if a speaker’s energized, walking around, using their hands, and projecting themselves, in-person’s just way better. You don’t get that feel from virtual classes.”*

*“[Polling] is a way to engage students without having individual pressure to answer, which I really like. And especially on Zoom, it makes it easier to get engaged with the material.”*

It should be noted that the flipped classroom approach and case-based interactive formats in both in-person and distance learning modalities emerged as clear favorites among many respondents. They reported that these approaches were preferred because they offered students the flexibility to engage with the materials at their own pace, effectively reinforcing key concepts and facilitating a deeper understanding of the subject matter.

*“I’m a huge fan of [the flipped classroom style]. As much as I like didactic, I really don’t like faculty spitting information at me. I’d much rather have the time to sit down, review it and then speak about it…I think flipped classroom is way more engaging…the material sticks a lot better too.”*

**Theme 3.**  *Distance learning was beneficial to students in many ways; however, the functional challenges cannot be ignored.*

Interview participants highlighted several benefits of distance education. They included access to recorded lecture materials for review, the ability to attend classes from home, reduced financial burden from commuting, greater flexibility with asynchronous sessions, attending remote lectures by guest speakers, and the freedom to take breaks as needed. Similarly, some participants also expressed a shift in perspective towards remote learning. Although initially skeptical, they discovered the convenience and benefits due to increased engagement and participation over time.

*“[I liked] not having to get ready or commute for an hour to school. I feel like [distance learning] enabled us to be able to have lectures from professors who may not have been able to make it… I think I ended up really liking it because it just gave you so much more flexibility.”*

In contrast, participants also brought attention to several challenges they encountered with remote classes. Those challenges included decreased engagement, screen fatigue, reduced motivation to participate, social isolation, and distractions from their environment or computer. They also expressed that it was harder to be seen or heard in larger online class settings. Notably, they pointed out challenges related to the social dynamics of distance learning.

*“I think for group learning or situations where… the teacher is asking a lot of questions; I think it’s definitely easier to be in-person and facilitates discussion easier and more people from the class talk and I think I have an easier time paying attention and holding my attention span for longer in an in-person situation.”*

The themes, subthemes, and illustrative quotes are also summarized and presented in Appendix A.

### 3.3. Mixed Methods Data

We used a Joint Display to compare quantitative and qualitative data. Table 2 is an integrated visual display that demonstrates how the survey results align or differ with qualitative themes.

## 4. Discussion 

Overall, our findings revealed the significant potential of distance education and acknowledged its diverse application to improve the learning experience in dental education. Our findings from three surveys over time indicate that at the start of the transition to distance learning, students initially exhibited negative and uncertain attitudes toward this approach. However, as they progressed towards in-person or hybrid learning, their perceptions of distance education became more positive. Our qualitative findings confirmed these results and indicated that this could be due to increased familiarity with distance learning, as well as increased faculty comfort in teaching online. 

Our findings also suggest that a hybrid learning model that combines remote didactics and in-person clinical instruction is a preferred approach. Our participants favored recorded live lectures as the most effective format for distance learning. Most survey respondents also indicated that between 25–50% of didactic courses should be delivered through distance learning. Our qualitative results further emphasized this observation. When participants provided a list of the didactic courses they felt were best delivered online, these were mainly non-procedure-based or non-clinical courses such as oral radiology, oral pathology, and basic dental sciences. Similarly, a research project carried out in two European countries, Denmark and Greece, indicated that a hybrid approach, particularly for preclinical theoretical subjects, is well-accepted among students [33]. 

Several studies have shown the importance of engagement in the success of distance learning. Previous studies found that engagement positively correlated with academic performance in distance learning environments [34,35] and highlighted the use of collaborative learning strategies to enhance engagement [36]. When asked about the most effective forms of participation in remote classes, students’ preferences were still diverse. Our participants have felt that nontraditional participation methods, such as annotating on a shared screen, participating in quizzes, and posting in discussion forums, are vital to engaging students. Moreover, their preference for speaking for participation increased during the pandemic, suggesting students’ comfort and adaptation to distance learning. This newfound appreciation for distance education reflected the adaptability and effectiveness of distance learning to meet the diverse needs of students. 

Our survey results suggest that distance learning had an impact on student’s burnout. However, there was a positive shift over time, with fewer students reporting an increase in burnout and more reporting no change or a decrease during the later pandemic years. Similarly, our results demonstrated that perceptions regarding distance learning’s effect on retention of class material improved over time, with fewer students reporting a decrease and more reporting no change in the later surveys conducted. Our qualitative data supported this, and students added context that this could be because they were able to review recorded lectures and hence were able to focus more on the lecture. The additional resources provided by distance education seem to complement and enhance students’ learning experiences without altering their preferred learning styles. In contrast, a few survey participants reported that their levels of engagement, retention, and burnout did not improve and may have worsened when compared to their pre-distance learning experiences. These findings suggest that while distance learning has many benefits, it may not be the optimal approach for every student. By understanding each student’s preferences and strengths in various learning settings, educators can promote a more inclusive and personalized curriculum. 

Our qualitative findings support additional strengths and limitations of remote learning that were identified through the surveys. The ability to attend the classes from their own homes was highly valued, as it provided a flexible and comfortable learning environment. The flexibility offered by remote learning empowered learners to manage their study schedules more effectively. Additionally, participants appreciated the opportunity to learn from national and international guest speakers who joined remotely, enriching the educational experience. 

Conversely, the limitations of remote learning were often related to students’ learning environment. They reportedly faced challenges such as distractions from their home environment and internet connectivity issues. Another significant aspect highlighted by participants was the impact of distance education on the social dynamics of learning. The absence of physical classroom interactions diminished the social aspect of education [37], impacting their relationships with instructors and peers, which is a crucial aspect of dental education [38,39]. The hands-on approach created a dynamic learning environment that encourages active learning experiences, promotes a deeper understanding of clinical subjects, and fosters students’ mental health [40]. Faculty should carefully plan the extent of distance learning incorporated for each course, considering the course subjects, overall class performance, and student mental and physical well-being. These limitations should be addressed to improve the experience of distance education.

Dental education is currently facing a faculty workforce shortage. The number of full-time faculty vacancies has increased, while the number of first-year student enrollees has grown, suggesting the likelihood of an even more severe student-to-faculty ratio issue [41]. In response, dental institutions must proactively assess and prepare to address potential gaps in the workforce. Our study suggests that distance learning may fill this gap by enabling students to learn from faculty who are not able to attend classes in person. Additionally, distance learning provides the opportunity to record sessions, hold didactic sessions with a large group of students, and utilize tools such as small group discussions to address the shortage of faculty not just nationally but globally. This finding is consistent with previous research suggesting that distance learning can help address faculty shortages in various academic settings [42,43].

The limitations of this study include the single-center design and the varying response rates of the surveys. Conducting the study at a single institution with a small class size may restrict the generalization of the findings. In addition, the varying response rates of the surveys could have influenced the results. The first survey was administered during a feedback session of the first course that utilized the remote module. It may have prompted a higher completion rate due to its novelty and timing during the early stage of the pandemic. In contrast, the second and third surveys were distributed after students returned to the school for in-person clinical learning. These surveys included four classes from each year, aiming to improve the sample size and obtain more comprehensive insights. However, reaching a wider group of students potentially resulted in lower response rates as students with busier academic and clinical schedules may have been less inclined to participate. Future research with larger sample sizes and multi-center designs, encompassing dental institutions with various class sizes, could further enhance our understanding of this topic. 

Despite these limitations, the results of this study contribute valuable observations to the existing knowledge on dental education and distance learning. To our knowledge, this is the first study of its kind that assesses dental students’ experiences with distance learning, utilizing a mixed-methods approach over a multi-year period. Through our study, educators can learn students’ perception shifts over time, transitioning from a more negative view to a more positive one regarding distance learning. Students’ positive and constructive feedback regarding distance learning has been highlighted as strategies to improve distance education. The findings also serve as a guide for further research and improvements in the dental curricula, leading the way for more effective and inclusive educational approaches.

## 5. Conclusions

Although challenges emerged during the COVID-19 pandemic and the transition between distance and in-person learning, students demonstrated adaptability and a willingness to embrace distance learning. We evaluated the perceptions of predoctoral dental students regarding distance learning throughout the pandemic, employing a mixed methods approach. As the situation was upheld, students’ attitudes shifted from uncertain and negative at the initial phase of remote learning to a more positive outlook as they adjusted to the new learning environment, even after transitioning back to in-person learning. Among various distance learning modules, recorded live lectures emerged as the preferred choice among students. Most students perceived that distance learning was better suited for didactic content, while it was considered less optimal for clinical subjects. Additionally, the majority of participants believed that distance learning would remain effective in the post-pandemic era. They also suggested that a hybrid learning approach, combining both in-person and distance learning, holds the key to the most effective educational experience moving forward. However, further research is necessary to assess the overall effectiveness in comparison to traditional methods, the social dynamics of distance learning, and how students’ perceptions of distance learning translate into academic and clinical performance.

## Figures and Tables

**Table 1:** Participant overview characteristics and response rates for three surveys.

	Survey Year			Qualitative Interviews
	2020	2021	2022	
Classes (% (n))				
2021	0% (0)	0% (0)	0% (0)	0% (0)
2022	100.00% (39)	32.00% (8)	34.48% (10)	27.27% (3)
2023	0% (0)	56.00% (14)	10.34% (3)	45.45% (5)
2024	0% (0)	12.00% (3)	27.59% (8)	18.18% (2)
2025	0% (0)	0% (0)	27.59% (8)	9.09% (1)
Response rate (% (n))	100.00% (39)	24.30% (25)	21.20% (29)	N/A (11)
Survey tool used	Zoom polling	Qualtrics	Qualtrics	Zoom

**Table 2:** Triangulation of data from quantitative and qualitative findings.

Key Research Questions	Preferred Learning Modality	Changes in Perspectives over Time	Impact on Learner’s Engagement with Content
Quantitative Data	Most students believe 25–50% of didactic material should be held via distance learning platforms in the future (64.81% agreement)	Although most students experienced decreased retention and increased burnout in the early pandemic, there was a positive shift over time.	Intellectual engagement remained the same or slightly decreased over time.
Qualitative Theme with illustrative quote	As found in theme 1 described above, the type of content influenced the student’s preferred modality. Students preferred in-person lectures for clinical topics and distanced lectures for didactic courses. “Hybrid is really my favorite. The reason I like Zoom classes is because I gain so much more time and it’s just easier on my schedule. But then, in-person classes are so much easier to follow and engage in. So, hybrid works best for me because too much in-person can just take so much time, and when I have one hundred percent Zoom classes, I just end up falling asleep in my bed.”	As discussed in theme 2, burnout was linked to factors including increased screen time, reduced socialization, and isolation, while decreased retention was attributed to fatigue. Over time, likely due to increased faculty and student comfort with the online platform, students recognized the benefits of distance learning, including the ability to review recorded lectures and engage more effectively through the use of annotation tools and technology. “I think at first, I was a little worried about it because just [distance learning] was new and everything was new, but I think it transitioned pretty smoothly and I got really used to it, and then it’s definitely fluctuated over time. I think when we started going back in person, I was really excited about that, but then after having a lot of in-person, I definitely miss the online aspect.”	In theme 3, we highlighted the positive aspects of distance learning that students valued, including access to diverse speakers, increased flexibility, reduced commuting time and costs, and the ability to review lectures at their convenience. However, students also expressed challenges like screen fatigue from prolonged computer use and reduced motivation to participate. Students described feeling hesitant to unmute and speak up in an online class and were less inclined to engage unless actively called upon. “it’s harder to engage [online]. It’s easier to be distracted. But when you have a small group, I feel like those cancel out, so I don’t have issues with distraction anymore and I’m more engaged”
Inferences	Most participants prefer a hybrid model consisting of a mix of distance and in-person learning, for didactic courses, seem to be the overall preference. In the qualitative interviews, participants explained that non-clinical didactic material is best suited for distance formats, while clinical-based subjects are best in person. There does not seem to be a single best option that fits all courses in the dental curriculum.	Students’ educational experiences of distance learning shifted from uncertain and negative to neutral and positive. The use of appropriate technology and teaching tools facilitated the improvement of student retention and reduced burnout.	In exploring our survey findings, from our qualitative data, we were able to identify that low engagement stemmed from students’ reluctance to actively participate in the distance learning setting, with many indicating greater comfort in speaking up in smaller groups such as in a breakout room.

## Data Availability

The data presented in this study are available on request from the corresponding author.

## Appendix A

**Table A1:** Summary of Qualitative Data Themes, Subthemes, and Quotes.

Theme I: Type of content influenced the student’s preferred modality	
Subtheme	*Quotes*
Distance formats were preferred for non-clinical didactic lectures, while in-person learning was preferred for clinical subjects.	“for lectures, I prefer the online format, because I think just having the home set up just makes me feel like I’m able to focus a little bit better, but then when it comes to the things that are more interactive, I definitely prefer the in person.” “[For] didactic courses, which we had a lot of in the first year, I preferred the online learning method because it is a lot of information and it’s really difficult to keep up with it in-person.” “I think more clinical content is better learned in-person. For example, when we were online learning a lot more biological science information and things like that, I think that’s perfectly fine to be virtual, because there’s less room for conversation about clinical cases and things like that compared to being in-person.”
Impact on learning styles	“As far as study habits, I think there weren’t any major shifts, but the big shift was that I was able to utilize the recordings and stuff. So yeah. I think my study times didn’t change too much. It was just maybe the resources that we had available were kind of different.”
Theme II: Appropriate use of technology and teaching tools helped facilitate learning in any modality	
Use of appropriate technology and teaching tools	“in terms of having a large classroom, online would be better because in an in-person setting, it’s so hard to have so many discussions going on at once because you’re in a large auditorium, everyone’s talking. No one can hear each other. Whereas with online, you can divide everyone into small groups of three or four, which is very effective on Zoom.”
Lecturer’s comfort and teaching style plays a significant role	“so much more fun when they can keep you engaged in the material and their jokes land a lot better because they’re hearing people laughing at it instead of just complete silence of their laptop screen. I like that aspect. The interactiveness is a huge plus in in-person lectures for me.”
Theme III: Distance learning comes with many benefits as well as functional challenges	
Benefits of distance learning	“I think with the online classroom, it’s easier to participate if you’re shy, I found, because in class, I think it’s usually the same people. There’s the chat feature. That’s a big one. I think people who never participate in class, I’ve noticed that they do participate in the chat. But in terms of keeping your attention, I would say in-person.” “oftentimes I find myself, if I’m in an in-person lecture, either getting distracted because the lecture isn’t moving fast enough or getting frustrated and giving up because it’s moving too fast. And I think that if you’re just going to give me a straight lecture, I would rather be able to pace myself in a way that matches my optimal learning speed. I think that being able to pause and really absorb the material helps. Or if a professor says something that I don’t understand, I can go back and rewatch it, and process it a little bit more before asking a question.”
Change in perception of distance learning	“I was never a fan of online courses because I was terrible at keeping up with deadlines and I liked having a set schedule. But then I think as it went on, I really liked being able to get up and not having to get ready or commute for an hour to school. I feel like it enabled us to be able to have lectures from professors who may not have been able to make it to class for whatever the reason, whether it was health issues or travels or whatever. That was nice because then we saw a variety of people we may not have seen before. So, I think I ended up really liking it because it just gave you so much more flexibility.”
Challenges faced during distance learning	“I feel like online; it just feels disconnected. Asking questions is difficult. Material just doesn’t feel like it sinks in as much, while in-person, you get a little bit more emotion from faculty. Questions are better. People are more engaged. Additionally, being online, you can also get really distracted easily.” “many of my faculty have a difficult time [navigating distance learning technologies]. It’s now years into the pandemic… and still we find ourselves walking faculty through Zoom… and it’s very tiring, but it is something that we’re happy to do, of course.
